# Supplementary material for: Impacts of land-use management on ecosystem services and biodiversity: an agent-based modelling approach
Source: PeerJ. 2016 Dec 22;4:e2814. doi: 10.7717/peerj.2814 (PMC5182993; doi:10.7717/peerj.2814)
Supplement: Supplemental Information 1 [file peerj-04-2814-s001.pdf]

Impacts of land-use management on ecosystem services and biodiversity: an agent-based modelling approach

Article S1: Model Documentation

Thomas J. Habib[1], Scott Heckbert[2], Jeffrey J. Wilson[3], Andrew J. K. Vandenbroeck[4], Jerome Cranston[1], and Daniel R. Farr[1]

[1]Alberta Biodiversity Monitoring Institute, Edmonton, Alberta, Canada
[2]Alberta Innovates Technology Futures, Edmonton, Alberta, Canada
[3]Green Analytics, Guelph, Ontario, Canada
[4]Silvacom Limited, Edmonton, Alberta, Canada

Corresponding author:
Thomas J. Habib[1]
Email address: thabib@ualberta.ca

Contents

1. Model and Landscape Set-up 2
2. Forest Timber & Carbon Model 5
Timber Cost Distance 9
3. Pollination Model 14
4. Biodiversity Model 16
5. Water Purification Model 18
Water Model Calibration 27

1. Model and Landscape Set-up

This process defines all variables, brings in GIS data, and includes model-wide user controls. Table A includes descriptions of all general and landscape variables; model-specific variables are presented in the appropriate model section below. An image of the model interface is presented in Figure A.

Table A. Global and cell variables describing the landscape and general model set-up.

Variable Name	Description	Units
User-defined & Imported Variables		
Global Model set-sup and control variables		
**session_string**	Optional descriptive suffix to append to all output files	text
**area**	Area of each cell	ha
**model-years**	User-defined duration of model simulations. Set on interface.	years
**discount-rate**	User-defined discount rate used in Net Present Value calculations. Set on interface.	%
**(variable)-dataset**	GIS raster input files for each landcover or other variables imported from GIS layers	.asc file
Cell Landcover Variables	Each variable represents the area of a landcover type within each cell in m^2. These variables are called by all models.	
**lc_20**	Natural water bodies including lakes, ponds, rivers, etc.	m^2
**lc_31**	Permanent snow cover (e.g. glaciers)	m^2
**lc_32**	Bedrock, talus, etc.	m^2
**lc_33**	River banks, bare soil, mud flats, etc.	m^2
**lc_34**	Disturbed land not captured in other Human Footprint categories	m^2
**lc_50**	Shrub-dominated land with <10% trees	m^2
**lc_110**	Predominantly native grasslands	m^2
**lc_120**	Agriculture	m^2
**c-p**	Forest with >75% conifer cover, at least half of which is pine	m^2
**c-s**	Forest with >75% conifer cover, at least half of which is spruce	m^2
**d**	Forest with >75% broadleaf cover	m^2
**mx-p**	Forest where neither conifers nor broadleaf cover make up 75% of the forest. At least half of all conifer cover is pine.	m^2
**mx-s**	Forest where neither conifers nor broadleaf cover makes up 75% of the forest. At least half of all conifer cover is spruce	m^2

**ag-qs**	Area of agricultural land in a cell	m^2
**for-qs**	Area of forest cutblocks in a cell	m^2
**hard-qs**	Area of hard linear features in a cell (e.g. paved roads)	m^2
**soft-qs**	Area of soft linear features in a cell (e.g. pipelines and transmission lines)	m^2
**urbind-qs**	Area of urban & industrial features in a cell	m^2
**hwet-qs**	Area of aquatic human footprint features in a cell (e.g. canals, reservoirs, tailings ponds)	m^2
**Pasture10**	Area of hay/tame pasture in a cell, based on a 2010 crop map. Subset of ag-qs	m^2
**canola-(year)**	Area under canola cultivation in a year. Subset of ag-qs	m^2
**Landscape descriptors**	Landscape characteristics of each cell that are not area measures	
**is-land-patch**	True/false variable indicating if the cell exists within the landscape (as opposed to another area of the rectangular modelling space outside the study region)	True/false
**natural-region**	Code (1-6) indicating the natural region of Alberta the cell falls within. 1 = Rocky Mountain; 2 = Foothills; 3 = Grassland; 4 = Parkland; 5 = Boreal; 6 = Canadian Shield	Categorical
**ecozone**	Code (1-6) indicating the ecozone the cell falls in. 1 = Boreal Plain; 2 = Montane Cordillera; 3 = Prairie; 4 = Taiga Plain; 5 = Boreal Shield; 6 = Taiga Shield	Categorical

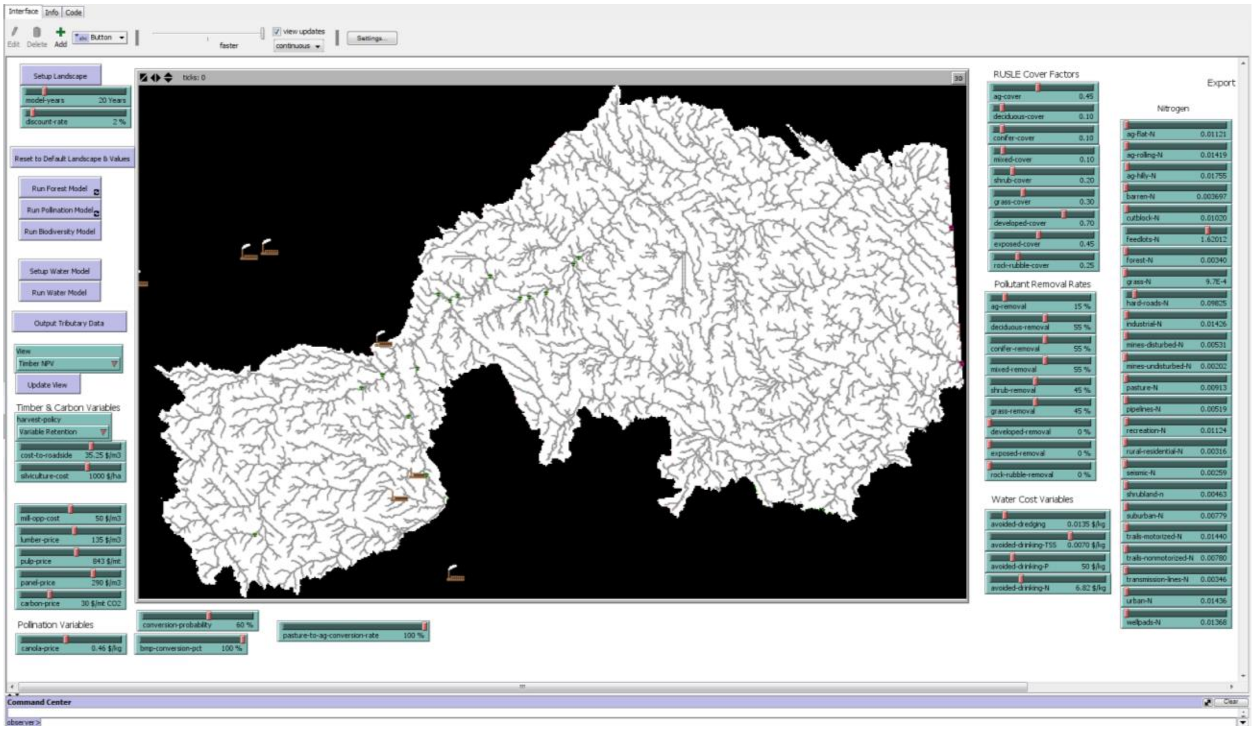

Figure A. Model interface using the NetLogo modelling platform. The North Saskatchewan watershed region of Alberta, Canada, is depicted.

2. Forest Timber & Carbon Model

This model has been developed to simulate forest growth and carbon storage in Alberta's Green Area, as well as estimating the economic value of harvested timber and stored carbon. Critical input landcover data include forest stand type and age, areas ineligible for timber harvest (e.g. riparian buffers), the location of mills, and a cost surface representing the cost to transport timber from forest stands to mills for processing. The model operates at an annual time step, and each step involves several sequential processes.

1. Forest growth

Forest growth is based on standardized yield curves (as a function of stand age) developed for each of 5 forest types (spruce-dominated coniferous; pine-dominated coniferous; spruce-dominated mixedwood; pine-dominated mixedwood; and deciduous) in each of the 2 primary forested ecozones in Alberta (Boreal Plains and Montane Cordillera), for a total of 10 forest strata in the model. Growth curves were obtained from Natural Resources Canada (2006). The percentage of coniferous vs. deciduous tree volume in each stratum, as well as initial stand ages, were obtained from ABMI's enhanced vegetation layer, which in turn is based on Alberta Vegetation Inventory (AVI) and provincial forest fire records. Further, each forest cell is defined as either eligible or ineligible for harvest, based on standard criteria for Alberta (parks, other protected areas, and riparian buffers are ineligible).

2. Timber harvest

The study area is divided into Forest Management Agreement areas (FMAs) that are managed by different forestry companies, and the locations of mills present in Alberta today are included in the model. In doing so, the model accounts for the spatial and temporal patterns of timber production costs and revenue. Each mill is assigned an area of land) from which to harvest timber (i.e. the appropriate FMA), a production capacity level (based on this historical public record), a mill type (sawmill, pulp and paper, oriented strand board, or mixed), and a preferred timber type (conifer or deciduous). The harvest algorithm asks each mill to examine its allocated forest area and harvests stands, prioritizing the stands > 80 years of age that have the least cost distance to the mill (See

Timber Cost Distance below). The annual allowable cut (AAC) for each FMA is allocated to its mills, and mills continue to harvest timber until the AAC is reached, less a user-defined percentage to account for the fact that actual wood harvest tends to be less than total AAC, based on historical records (AESRD, 2013). Once harvested, the stand age of each harvested cell is set to zero, and the adjusted harvested volume is sent to the mill.

3. Economic valuation of timber

Timber value is defined as the market value generated from a given volume of timber; that is, timber value is equivalent to the profits generated at the mill from selling timber products. Net profits for each mill are defined as mill revenue, minus timber delivery and operational wood processing costs. Mill revenue is determined based on the volume and type of wood products (including lumber, pulp and paper, oriented strand board, or veneer) typically produced at each mill. Provincial 1-year results for timber revenue are provided inTable B. The profits from each mill are then mapped back to the landscape, based on the amount of wood that flowed from each cell to the mill in a given time period.

4. Model Carbon Storage and Economic Value of Carbon

Forest carbon storage-versus-age curves were defined for each of the 10 forest strata listed above (5 stand types in each of 2 ecozones), based on a published forest carbon model (CBM-CFS3; Kull et al. 2011; Kurz et al. 2009). The process adopted was as follows: First, forest strata yield curves were entered in to the CBM-CFS3 model for each ecozone. Second, a stand level simulation is run for each stratum-ecozone combination. After a 200 year simulation, the model outputs a series of carbon stock data for each carbon pool (in tonnes per ha) for each stratum within each ecozone. From this output data, a series of “carbon yield” equations are developed by regressing carbon stocks against stand age (See example for two carbon pools in Figure B). These relationships are then coded in NetLogo so that carbon storage is tracked for each forest stand as they age. The model tracks 9 separate pools, which are subsequently grouped into the 5 pools used by the Intergovernmental Panel on Climate Change (IPCC) for reporting purposes (Table C).

The economic value of stored carbon is based on a per-tonne price for CO_2-equivalent, which is set by the user. The model calculates both the total value of stored carbon in forested areas, as well as the change in carbon storage over the model simulation period.

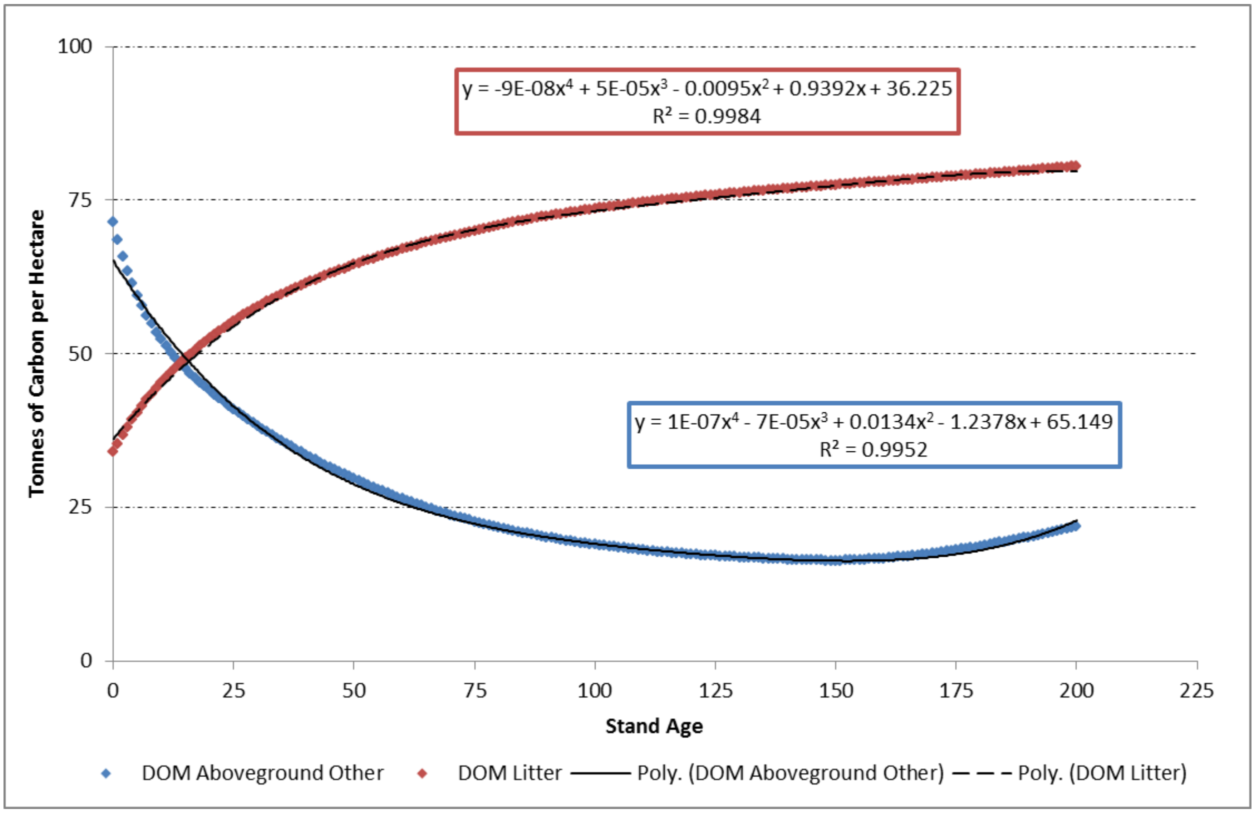

Figure B. Example of carbon storage vs. stand age for two carbon pools, generated using the CMB-CFS3 model.

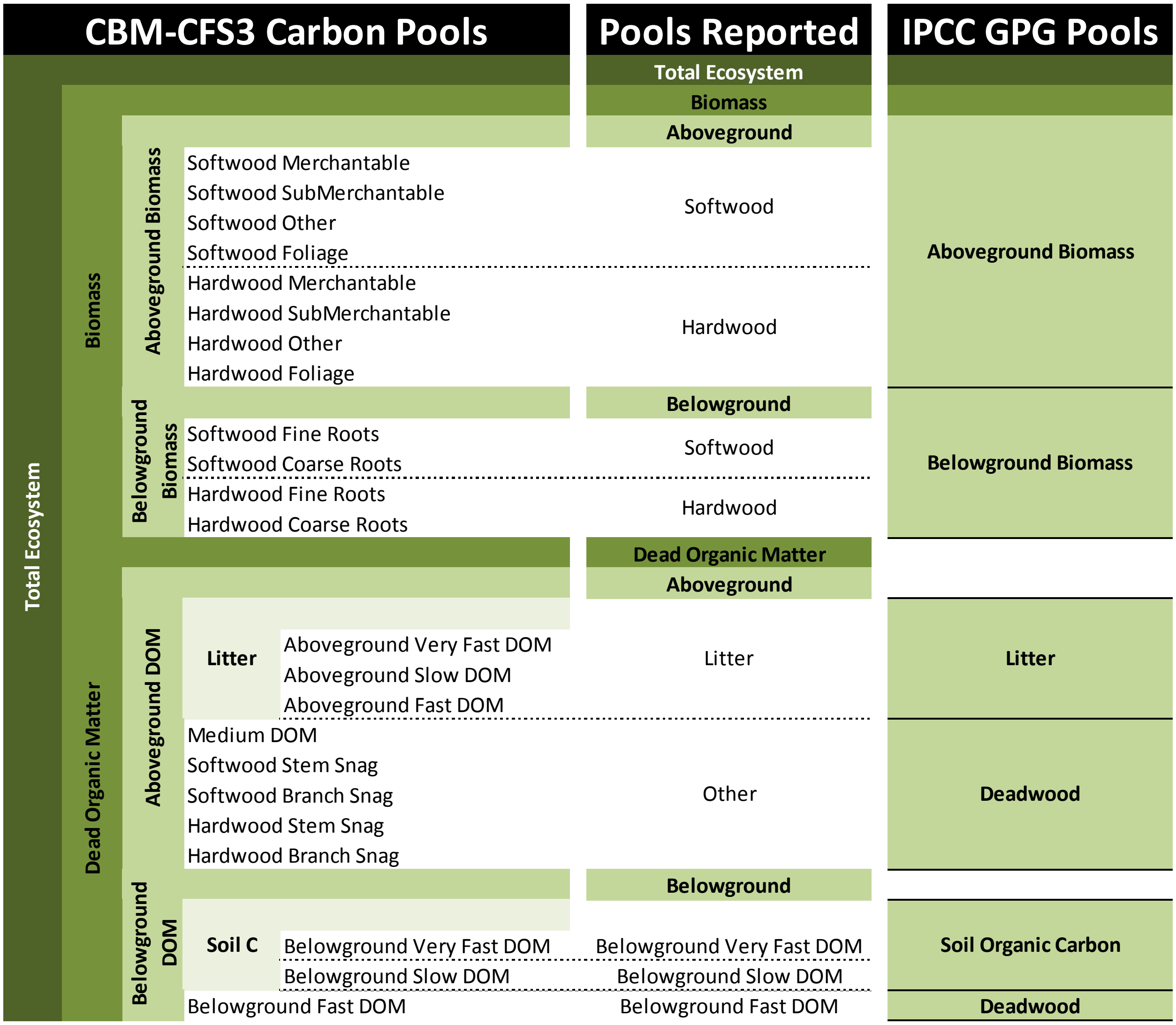

Figure C. Carbon pools from the original CBM-CFS3 model, grouped into pools reported in the ESA model, and how they correspond to the carbon pools used by the IPCC.

Table B. One-year modelled timber revenues by watershed, based on 2010 landcover and wood product prices.

Watershed Region	Estimated 1-Year Revenue
North Saskatchewan	$164M
South Saskatchewan	$17.3M
Red Deer	$39.9M
Upper Athabasca	$535M
Lower Athabasca	$378M
Upper Peace	$1.35B
Lower Peace	$837M
**TOTAL**	**$ 3.32B**

Timber Cost Distance

Each mill has its own cost distance raster, representing the estimated cost per unit volume to transport timber from each cell to the mill. All cost distance rasters are generated from a single cost surface, which represents the cost to transport timber across each cell (in $\$/m^3$). The cost to transport timber is based on a fixed hourly rate of $130/hr, therefore the main factor is travel speed (Joerg Goetsch, pers comm.). The $130/hr rate coverts to a per-km cost for a truckload at different travel speeds; travel speeds were assumed for different types of roads (Table C).

For chips, there are 2.75 m^3 per BDT (bone dry tonne), 21 BDTs per truck, therefore 57 m^3 per tuck of hardwood chips (assume hardwoods are chipped at roadside). There is less volume per truckload of softwood, which is not chipped, so a correction factor of 1.623 is applied to the volume of softwood timber that is transported during this phase of the Forest Timber model.

For the cells that intersect road features, each cell was characterized by the length of the highest-speed road that occurs within it. For example, if a cell contains a paved road (speed = 90), it is assumed that the wood will travel along whatever the length of paved road lies within it, even if other lower-speed roads are present. Using the Cost Distance tool in ArcGIS 10.1, we generated a cost distance raster for each mill based on this cost surface (i.e. the cumulative cost of cells along the least-cost pathway from each forested cell to the mill in question). This value represents the monetary transportation cost to move timber from a cell to the mill for processing.

Table C. Characteristics of transporting timber along different road types.

Road Type	Road Speed km/h	Cost per load $/km	Cost per m^3 $/km
Paved roads	90	1.44	0.0253
Gravel 2-lane roads	70	1.86	0.0326
Gravel 1-lane roads	50	2.60	0.0456
Dirt roads	40	3.25	0.0570
Winter roads and truck trails	30	4.33	0.0760
No roads present (cutlines only)	-	-	1.00 (assumed)

Table D. Variables used in the Forest Timber & Carbon Model.

Variable	Description	Units
**Timber and Carbon Model**		
**Global variables and model control**		
**area-harvested-total**	Total forested area harvested by all mills	m^2
**harvest-pct-task**	Subroutine to quickly calculate harvest-pct for each harvested cell	n/a
**harvest-pct**	Percentage of a cell to be harvested if model set to "variable-retention"	%
**value-per-ha**	Calculates and tracks average value per ha of harvested patches	$ per ha
**npv**	Calculates and tracks the net present value timber harvest from all harvested patches	$
**npv-vol**	Calculates and tracks the total volume harvested associated with npv	m^3
**npv-per-m^3**	Calculates and tracks the average net present value per m^3 (npv/npv-vol)	$ per m^3
**Cost-to-roadside**	Assumed harvest costs to roadside, set by model user (default setting is $35)	$ per m^3
**Silviculture-cost**	Assumed silvaculture costs associated with harvested patches, set by model user (default setting is $1000)	$ per ha
**Mill-opp-cost**	Assumed mill operational costs to process wood into wood products, set by model user (default setting is $50)	$ per m^3
**Lumber-price**	Current lumber price, set by model user (default setting is $135)	$ per m^3
**Pulp-price**	Current pulp price, set by model user (default setting is $843)	$ per metric ton
**Panel-price**	Current panel price, set by model user (default setting is $290)	$ per m^3
**Carbon-price**	Current carbon price, set by model user (default setting is $15)	$ per metric ton CO_2
**Timber model landscape variables**		
**fmu**	fmu-dataset allocated to patches	as above
**age-class**	age-dataset allocated to patches	as above
**stand-age**	Represents stand age allocated to patches based on age-class	Age in years
**ineligible**	ineligible-dataset allocated to patches	as above

**is-forest-patch**	Variable taking the value of 1 or 0 used to set forest-patches agent set	True/false
**is-eligible**	Variable taking the value of 1 or 0 used to set eligible-forest agent set	True/false
**total-patch-volume**	Total volume for each patch as calculated based on yield curves	m^3
**conifer-volume**	Conifer volume for each patch as calculated based on yield curves	m^3
**deciduous-volume**	Deciduous volume for each patch as calculated based on yield curves	m^3
**my-patch-vol-harvested**	Calculates, sets, and stores the volume harvested from each patch	m^3
**area-harvested-from-this-patch**	Area of forest within a cell that is harvested	ha
**patch-haul-cost**	Cost to transport timber from a cell to the mill that has harvested its timber	$\$/m^3$
**is-harvested**	True/false indicating if a cell is harvested	True/false
**haul-cost-XXX**	Cost to transport timber from a cell to mill XXX (using “mill-namecode” to identify each mill)	$\$/m^3$
**timber-esv**	Calculates timber production value for each patch by multiplying npv-per-m^3 by my-patch-vol-harvested	$ per cell
**timber-evsp**	Calculates potential timber production (i.e. unharvested eligible forest volume) value for each patch by multiplying npv-per-m^3 by total-patch-volume	$ per cell
**Carbon Model Variables**		
**aboveground-soft**	Calculates above ground forest carbon from conifer (see appendix A)	Tonnes of Carbon
**aboveground-hard**	Calculates above ground forest carbon from decid (see appendix A)	Tonnes of Carbon
**belowground-soft**	Calculates below ground forest carbon from conifer (see appendix A)	Tonnes of Carbon
**belowground-hard**	Calculates below ground forest carbon from decid (see appendix A)	Tonnes of Carbon
**dom-above-other**	Calculates above ground carbon content of dead organic matter other than forest litter (see appendix A)	Tonnes of Carbon
**dom-above-litter**	Calculates above ground carbon content of dead organic matter from forest liter (see appendix A)	Tonnes of Carbon
**dom-below-slow**	Calculates below ground carbon content of slow decomposing dead organic matter (see appendix A)	Tonnes of Carbon
**dom-below-fast**	Calculates below ground carbon content of fast	Tonnes of

	decomposing dead organic matter (see appendix A)	Carbon
**dom-below-vfast**	Calculates below ground carbon content of very fast decomposing dead organic matter (see appendix A)	Tonnes of Carbon
**ipcc-above**	Regroups the carbon categories to match ipcc categories	Tonnes of Carbon
**ipcc-below**	Regroups the carbon categories to match ipcc categories	Tonnes of Carbon
**ipcc-litter**	Regroups the carbon categories to match ipcc categories	Tonnes of Carbon
**ipcc-deadwood**	Regroups the carbon categories to match ipcc categories	Tonnes of Carbon
**ipcc-soc**	Regroups the carbon categories to match ipcc categories	Tonnes of Carbon
**ipcc-total**	Sum of all ipcc carbon groupings	Tonnes of Carbon
**ipcc-start**	Total carbon at start of simulation	Tonnes of carbon
**ccs-year**	Change in carbon storage in a given model year	Tonnes of carbon
**ccs-total**	Change in carbon storage from the start to a given model year	Tonnes of carbon
**ccs-flux**	Change in carbon storage from start to end of simulation	Tonnes of carbon
**carbon-esv**	Stock of forest carbon in CO_2e for each patch multiplied by the carbon price (in $ per CO_2)	$ per patch
**ccs-esv**	Represents the carbon flux on each patch by calculating the carbon capture and storage for each period t	$
**Mill Agent Variables**		
**mill-id**	GIS data applied to mills attributing each mill an id number	Nominal
**mill-name**	GIS data applied to mills attributing each mill its operating name	Categorical
**mill-namecode**	Three-letter code for each mill. Used in “haul-cost-XXX”	
**mill-type**	GIS data applied to mills attributing each mill an operational type (Sawmill, Pulp mill, OSB mill, or some combination)	Categorical
**my-mill-patches**	A variable that defines where each mill can and cannot harvest in each period (defined as stand > 80 years and within the mills corresponding FMU)	A set of patches
**my-aac-sw**	GIS data applied to mill attributing each mill its	m^3 per year

	coniferous annual allowable cut	
**my-aac-hw**	GIS data applied to mill attributing each mill its deciduous annual allowable cut	m^3 per year
**patches-harvested-count**	A variable that allows each mill to track which patches it has during the current period	A set of patches
**my-harvested-mill-patches**		
**mill-area-harvested**	A variable that allows each mill to track how much area it has harvested during the current period	hectares
**sw-vol-harvested**	Conifer volume harvested in period t	m^3
**hw-vol-harvested**	Decid volume harvested in period t	m^3
**gross-merch-vol-sw**	Adjust the total sw-vol-harvested to account for only merchantable volume (assumed to be 92% of total volume)	m^3
**gross-merch-vol-hw**	Adjust the total hw-vol-harvested to account for only merchantable volume (assumed to be 92% of total volume)	m^3
**cumulative-vol**	A variable that allows each mill to track the total volume harvested across all time periods	m^3
**haul-cost**	Calculates the total haul cost for each mill by multiplying the distance-cost ($ per m^3 per km) by avg-dist-to-mill by and harvested volume in period t	$
**delivered-mill-cost**	Calculates the delivered mill cost in the current period (see section 3.6)	$
**mill-cost**	Calculates all mill costs in the current period (see section 3.6)	$
**mill-revenue**	Calculates the mill revenue from sale of forest products (see section 3.6)	$
**net-profit**	Calculates net profit (mill-revenue minus mill-cost) for each mill in the current period	$
**mill-npv**	Net present value earned by a mill over the simulation period	$

3. Pollination Model

The Pollination Model focuses on assessing the economic value added to canola production as a result of pollination by native bees. Using empirical relationships obtained from Morandin et al. (2006) in northwest Alberta, we parameterized a quarter-section-level model of canola yield as a function of bee abundance, which was in turn based on an empirical relationship with the amount of uncultivated land within neighbouring quarter-sections. Vector-based landcover and human footprint data obtained from ABMI were used to delineate the boundaries of crop fields and uncultivated areas. The advantage to using the vector-based ABMI dataset to delineate polygons is that they capture any small uncultivated parts of a field that can contribute pollinator nesting habitat that would not be recorded using a 30m resolution landcover raster.

In each time step of this annual model, the following processes are simulated:

1. Crop rotation

To obtain the location of canola fields, we used 4 years of annual crop maps (2009-2012; resolution 30m or 56m) developed by Agriculture and Agri-food Canada (AAFC 2012). These raster layers were used to determine the identity of crops within each agricultural polygon delineated by the ABMI vector landcover layer. In each year, the amount of each crop type in each cell changes based on these data, cycling through a 4-year rotation; after the 4th year, the rotation starts again.

2. Calculate bee abundance and canola yield

A bee abundance index (*BA*) in each cell containing canola was estimated with the following equation from Morandin & Winton (2006):

$$BA = 6.06 + 0.264NL$$

where *NL* represents the area of natural and semi-natural land (measured in hectares) within the target cell and the 8 adjacent neighbouring cells. This includes all landcover types undisturbed by humans, including pasture but not annual cropland.

The estimated bee abundance was then used to calculate the seed deficit *SD* as follows:

$$SD = -12.54 + 1.29BA^{0.48}$$

Seed deficit is a proxy for yield, representing the difference between a canola pod's maximum possible seed set with the observed seed set. Based on data provided by Morandin & Winston (2006), each additional seed per pod represents an increase in canola yield of 73.3 kg/ha. The difference between the estimated seed set with pollinators present and the expected seed set if all pollinators were removed represents the fraction of canola yield attributable to insect pollinators.

3. Calculate pollinator value

The additional yield attributed to pollinators is multiplied by the market price of canola seed to obtain the monetary value of pollinators in a given model year. For multi-year simulations, the model calculates the net present value of pollinators based on a user-defined discount rate. Provincial modelled results for the portion of canola profits attributable to pollinators, based on four years of crop rotation data (2009-2012) are provided in Table E.

Table E. Modelled pollinator value by watershed region over a 4-year period, based on crop maps from 2009-2012.

Watershed Region	Estimated 4-Year Pollinator Value
North Saskatchewan	$1.00B
South Saskatchewan	$343M
Red Deer	$449M
Upper Athabasca	$188M
Lower Athabasca	$79.5M
Upper Peace	$429M
Lower Peace	$163M
**TOTAL**	**$2.65B**

Table F. Variables used in the Pollination Model

Variable Name	Description	Units
Pollination Model		
**all-canola-patches**	Cells that contain canola at some point during the simulation	Set of cells
**canola-patches-this-year**	Cells containing canola during the current time step (year)	Set of cells
**ticks-xxxx**	Model time steps (ticks) corresponding to the xxxx year of a crop rotation.	List of year (aka "ticks")
**Canola-price**	Market price of canola	$
**natland-area**	Area of natural and semi-natural land (incl. pasture) in a cell. Calculated from GIS landcover variables.	m^2
**natland-buffer**	Area of natural land in a cell and its 8 adjacent neighbouring cells	m^2
**bee-abund**	Estimated bee abundance index in a cell	n/a
**poll-def**	Estimated deficit in canola seeds per pod (0 represents full seed set). Later converted to yield	# seeds
**bee-seedset**	The amount of seeds/pod attributed to pollinators	# seeds
**poll-yield-ha**	Canola yield per hectare, attributable to pollinators	kg/ha
**poll-profit-ha**	Economic value per hectare of yield attributable to pollinators	$/ha
**poll-profit-patch**	Economic value of yield attributable to pollinators	$
**pollination-npv**	Net present value of pollinators based on a multi-year simulation, based on a user-specified discount rate	$

4. Biodiversity Model

The biodiversity model estimates the effect of human land-use footprint on a variety of species. The biodiversity index, ranging from 0 – 100%, represents the difference between reference (i.e. "de-footprinted") and current abundance of each species, averaged across all species. The biodiversity model is a simplified version of ABMI's biodiversity intactness index (ABMI 2012) that is able to easily integrate with the other ecosystem service models.

ABMI's original biodiversity index analysis calculates intactness for each species as the ratio of current abundance to reference abundance (multiplied by 100%), or the inverse if the current abundance is greater than the reference abundance. Reference abundance is the predicted abundance of the species when the human footprint is set to 0 ("de-footprinted"). Current abundance is the predicted abundance at current human footprint levels. The same set of models is used for both predictions. The abundance metric for the biodiversity index is currently the probability of occurrence. Probability of occurrence is modelled as a binomial variable using a logit-link, with several trials per site (sample). Specifically, each site includes, 4 quadrats for plants, mosses, and lichens; 4 soil samples for mites; and 9 point counts for birds. Habitat elements are modelled as negative binomial counts with a log-link, a single-trial binomial with a logit-link for cover or log-normal, as appropriate for the different measures.

In addition to using footprint types as explanatory variables, the models also include covariates for vegetation types and geographic location. The habitat covariates for the boreal and foothills regions are currently vegetation types and ages of forest stands derived from ABMI's wall-to-wall vegetation mapping. Vegetation categories include pine, non-pine upland conifer (spruce), lowland conifer (black spruce/larch), deciduous, mixedwood, shrub, grass, non-treed wetlands, barren and water (the latter two are assumed to be 0). Age classes of forest stands are tracked by year and summarized into 20 year age classes, but combined into 3 broad groups in the modeling: 0-40yr, 41-100yr and 100+yr. In the grasslands and parklands, soil types are used as the habitat descriptors, because these are mapped even where agricultural footprint has replaced the native vegetation over large areas.

The current NetLogo model uses a simplified version of the species-specific intactness models, providing an estimate of how the overall biodiversity index (i.e. the average biodiversity index across all species) responds to footprint levels. We generated an equation to fit overall biodiversity index as a function of the percentage of different broad footprint types within a quarter-section. Models were developed for hard linear features (e.g. roads), soft linear features (e.g. seismic lines, pipelines, road margins), urban/industrial areas, agriculture, and forestry cutblocks. Each equation is of the form

$$\text{Quarter-section biodiversity index} = 100 + a*pcHF + b*\ln(pcHF+1) + c*pcHF^2$$

where pcHF is the percent (0-100) of the human footprint type in a quarter-section, and a, b and c are model coefficients specific to each footprint type. These fit the observed results very accurately, but they apply to individual footprint types. In quarter-sections containing multiple footprint types, the equation is used sequentially, from the most-severe footprint type to the least-severe type (in

descending order of severity, hard linear, soft linear, urban/industrial, agriculture, and forestry), in the following procedure:

1. First, the model uses the above equation to calculate intactness for the most severe HF type: hard linear. For example, if there was 5% hard linear footprint present in a quarter-section, the above equation, parameterized for the hard linear footprint type, would calculate intactness at 83.7%.
2. For the next-most severe HF type present, soft linear, add the amount to the amount of hard linear, and use the equation for soft linear to calculate how much intactness drops from the hard linear level to the hard linear + soft linear level. For example, if there is 10% soft linear (along with 5% hard linear), then the model calculates intactness at 5% soft linear and at 15% soft linear, which are 87.2% and 65.7%, respectively. The difference is 21.5%, which represents the incremental decrease in intactness attributable to the additional 10% soft linear footprint; thus, the overall intactness score is updated to 83.7% - 21.5% = 62.2% intact.
3. Step 2 is repeated sequentially for the remaining footprint types, in order of most- to least-severe (urban/industrial, followed by agriculture, followed by forestry). For example, if there was 20% urban/industrial footprint present in the same quarter-section, the model would use the urban/industrial equation at 15% and 35%, resulting in intactness values of 55.9% and 40.5%, for an additional drop of 15.4%. As above, intactness would update to 62.2% - 15.4% = 46.8% intact.

Based on a random sample of 10% (n = 11007) of all cells in the North Saskatchewan Watershed region, the simplified model fits the full model very well (r^2 = 0.94).

Table G. Variables used in the Biodiversity Model.

Variable Name	Description	Units
Biodiversity Model		
**hard-linear-intactness**	Intermediate biodiversity calculation after accounting for hard linear footprint	%
**soft-liner-intactness**	Intermediate intactness calculation after accounting for soft linear footprint	%
**urbind-intactness**	Intermediate intactness calculation after accounting for urban & industrial footprint	%
**agric-intactness**	Intermediate intactness calculation after accounting for agricultural footprint	%
**forestry-intactness**	Intermediate intactness calculation after accounting for forestry cutblocks	%
**intactness**	Final calculation of the biodiversity index	%

5. Water Purification Model

The water purification model represents selected hydrological processes in Alberta watersheds related to non-point source pollution through surface flow and erosion. In particular, the model was designed to identify source areas of pollutants, important areas for removing pollutants, and impacts to downstream water users. Note that this model only includes surface flow and run-off, and does not incorporate ground water flow. This dynamic model involves several processes:

1. *Model Set-up*
   This step brings in all the relevant spatial data, including landcover, precipitation, river network, and monitoring points of interest. All figures provided in this document depict the North Saskatchewan and Battle River Watersheds in Alberta, Canada.
2. *Precipitation and Surface Flow*
   A rainfall event is simulated, where the average annual amount of precipitation falls on each cell and runs off downslope to the river network, eventually flowing downstream to the river outlet.
3. *Pollutant Loading and Deposition*
   Pollutant loading is based on nutrient export coefficients (N, P, TSS) or RUSLE (sediment) combined with annual precipitation. Deposition is based on landcover types.
4. *Stream Flow & Tracking Loads*
   The flow of surface water, nutrients, and sediments through the river network is modelled to calculate the cumulative annual load of each pollutant throughout the river network.
5. *Source and Sink Areas*
   After overland and stream flow are complete, nutrients and sediment that reach the river network can be traced back to their origin points on the landscape.

1. Model Set-up

A river network for a major watershed was created using the flow accumulation tools in the Spatial Analyst (Hydrology) toolset in ArcGIS, based on a DEM of Alberta (Figure D). The resulting network was visually compared to a GIS layer of streams in Alberta to assess its accuracy; any incorrect river connections caused by importing a linear river layer at an 800m resolution were manually corrected. This layer is imported into NetLogo such that any 800m cell that intersects a river is classified as a “river” cell. A network is created so that each river cell tracks its upstream and downstream neighbours, allowing for water and information to travel throughout the network. Finally, water monitoring stations are created to facilitate tracking of water flow and quality metrics at points of interest, such as mouths of tributaries, inlets of important lakes, river outlets, and municipal water treatment plants.

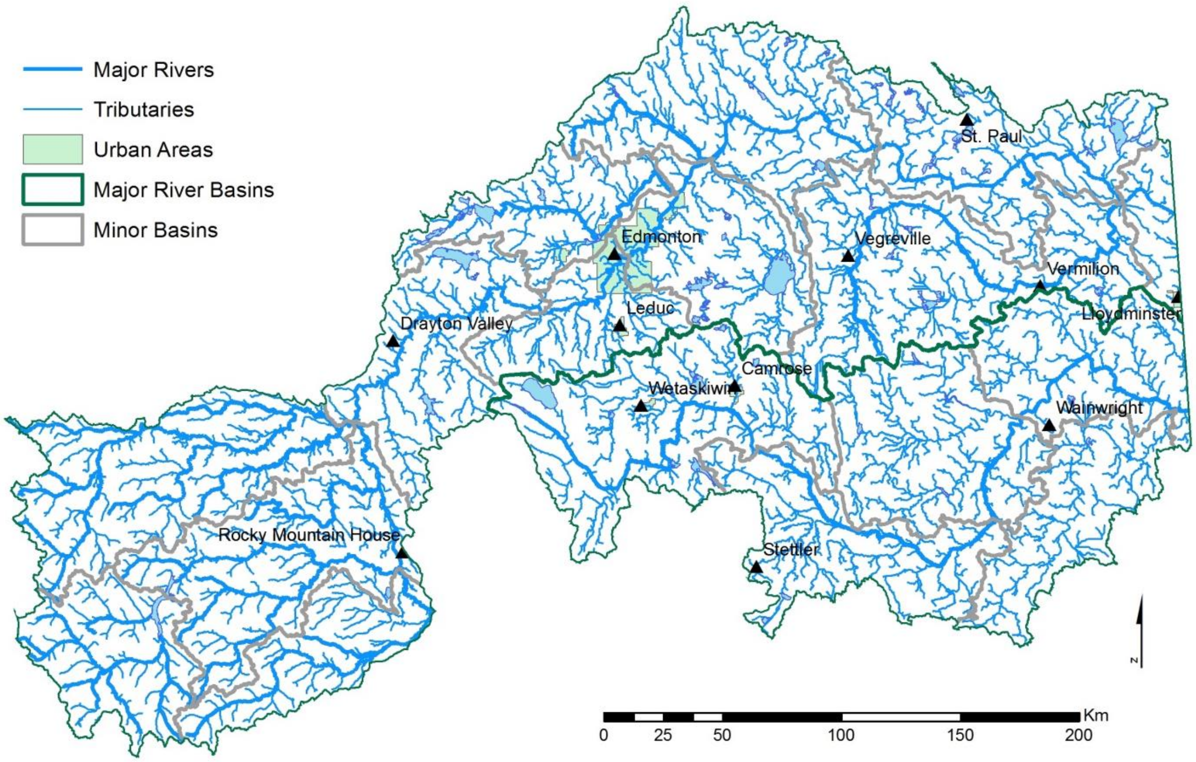

Figure D. North Saskatchewan and Battle River Watersheds and river networks in Alberta, Canada.

2. Precipitation and Surface Flow

Precipitation is based on mean annual precipitation across Alberta, and is represented as a set of "raindrop" water agents with a specified volume of water, corresponding to mean annual precipitation falling on each patch of land. Runoff for each patch is calculated as a percentage of total precipitation, based on runoff coefficients for each landcover type (Table H; Donahue et al. 2013). Because each cell contains multiple landcover types, each cell's runoff coefficient is calculated as the area-weighted average of runoff coefficients for each landcover category. After the precipitation event, the volume of water that runs off each patch of land moves to the adjacent patch with the lowest elevation, and this downslope movement continues until each raindrop agent reaches a river cell.

Table H. Runoff coefficient for Alberta landcover types (Donahue 2013).

Landcover Type	Runoff Coefficient
Agriculture	0.5
Forest	0.3
Shrubland & Cutblocks	0.28
Grassland & Pasture	0.26
Hard Linear Features (Paved & Gravel Roads)	0.89
Soft Linear Features (Trails, Seismic lines, Pipelines, Transmission Lines)	0.5
Wellpads, Barren, Urban, and Industrial Areas	0.69

3. Pollutant loading and deposition

Nutrients (nitrogen, phosphorus, and total suspended solids – TSS) and sediment are loaded into surface water flow through two different modelling processes, outlined below.

Water Quality

Nutrients (nitrogen and phosphorus) and TSS are loaded into water flow based on export coefficients (measured in $kg \cdot ha^{-1} \cdot mm^{-1}$ of annual precipitation) calculated for major landcover and human footprint types (Donahue 2013). Each 800m cell in in the model includes multiple landcover and human footprint types, so pollutant loading is calculated as an area-weighted average across each landcover type present within a cell. Thus, the amount of pollutant k initially loaded into raindrop agent *j* (P_{jk}, measured in kg) at origin cell *c* is calculated as:

$$P_{jk_c} = \sum_{L} E_{kl} \cdot A_l \cdot W$$

Where *L* is the number of unique landcover types in the origin cell of the raindrop *j*, E_{kl} is the export coefficient for pollutant *k* in landcover type *l* (measured in $kg \cdot ha^{-1} \cdot mm^{-1}$ of annual precipitation), A_l is the area of landcover type *l* within a cell (measured in ha), and *W* is annual precipitation (measured in mm).

Nutrient removal occurs during overland flow, where a percentage of each raindrop agent's pollutant load is removed as it flows across downslope cells before reaching the river network. Pollutant removal percentages are assigned for each landcover type, and the total amount removed by a cell is the area-weighted average of the landcover types comprising the cell. Thus, the amount of pollutant *k* in raindrop agent *j* after it has passed through adjacent cell *c + 1* is calculated as:

$$P_{jk_{c+1}} = P_{jk_c} \cdot \sum_{L} D_l \cdot A_l$$

Where D_l is the percentage of each pollutant removed from landcover type *l*. This process is repeated as the raindrop agent passes through all downslope cells until it reaches the river network. Example maps are provided for phosphorus loading (Figure E) and removal (Figure F).

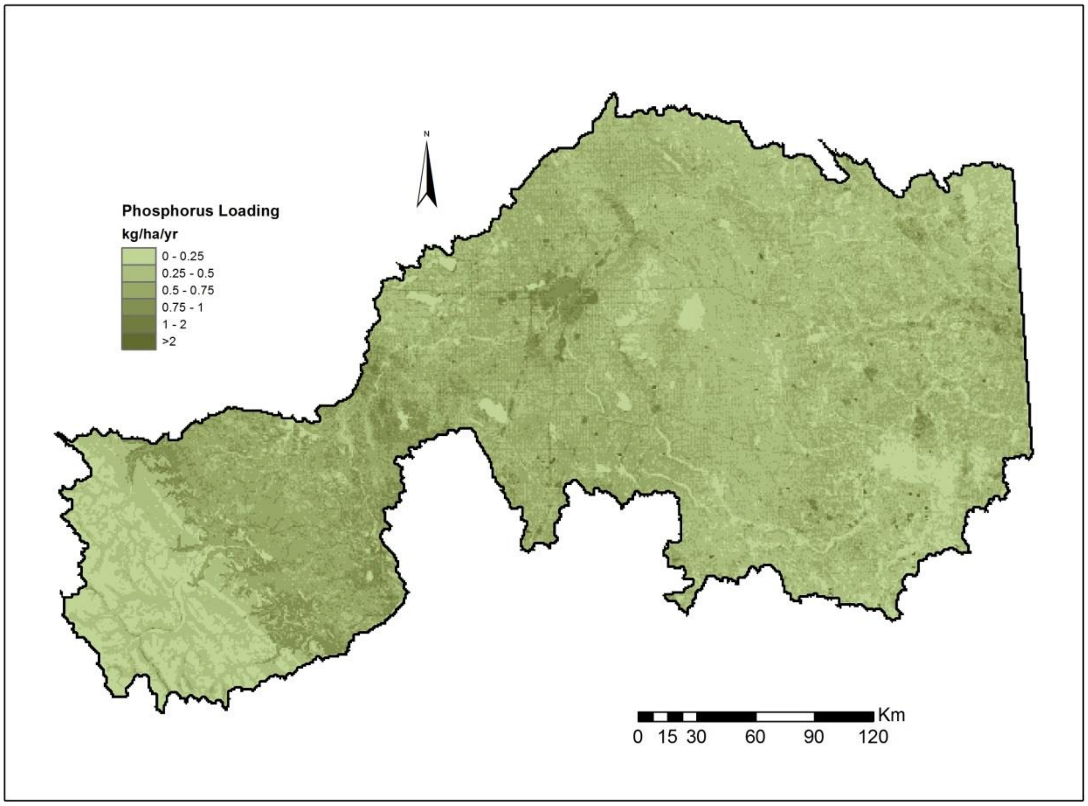

Figure E. Modelled phosphorus loading into surface runoff in the North Saskatchewan watershed region of Alberta, Canada, based on average precipitation from the 1971-2001 climate normals.

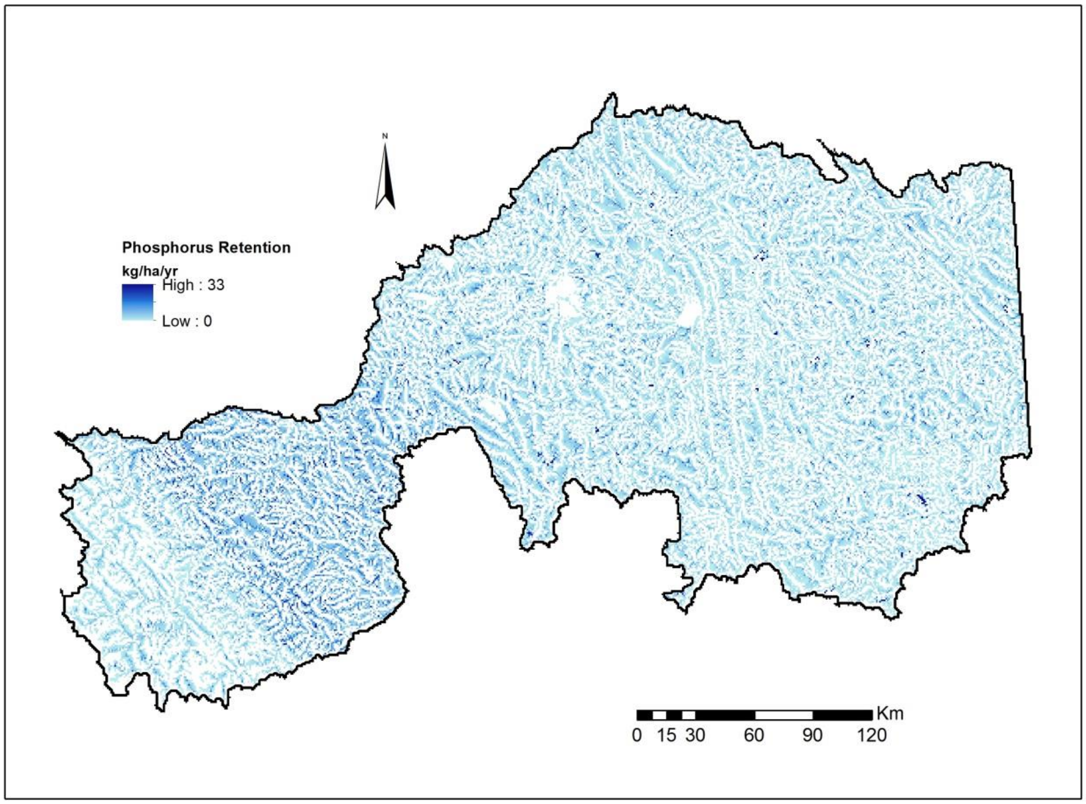

Figure F. Phosphorus retained per 64 ha cell in the North Saskatchewan and Battle River Watersheds in Alberta, Canada.

Sediment

Sediment erosion into surface water flow is calculated using the Revised Universal Soil Loss Equation (RUSLE; Renard et al. 1997). Sediment erosion from each patch is calculated (in tonnes) as

$$Sediment = R \cdot K \cdot LS \cdot C$$

Where *R* is the rainfall erosivity factor, *K* is the soil erodibiity factor, *LS* is the slope factor, and *C* is the area-weighted cover factor that accounts for the influence of landcover types (e.g. vegetation, human use, etc.) on erosion. The R value was set at 0.350, following guidance from Wall et al. (2002). The K factor was based on soil texture categories (Wall et al. 2002); soil information was obtained from the Soil Landscapes of Canada version 3.2 (REF). The LS factor was calculated based on a digital elevation model using the Terrain Analysis tools in SAGA GIS (Bohner & Selige 2006). The C factor was based on

generalized C values for Alberta generated by Wall et al. (2002). We calculated the area-weighted C-value for each cell as

$$C = \sum_{L} C_l \cdot A_l$$

Where C_l is the cover factor for landcover type l, A_l is the area of landcover type l within a cell, and L is the number of distinct landcover types within a cell. In addition to these terms, RUSLE typically includes a term for the support practice factor (P), which represents practices undertaken to modify runoff such as changing grade, flow pattern, or the direction of runoff. Common examples of support practices include cross-slope cultivation, contour farming, stripcropping, and terracing (Wall et al. 2002). P values represent a percentage reduction in erosion, and range from ~0.1 (90% reduction in erosion) to 1.0 (no change) if no support practices are employed. Given the lack of spatially explicit data on support practices in Alberta, this term was omitted in the present model (corresponding to a P factor of 1.0).

An example output map of sediment generation is depicted (Figure G). Sediment deposition occurs via the same process as removal of water pollutants.

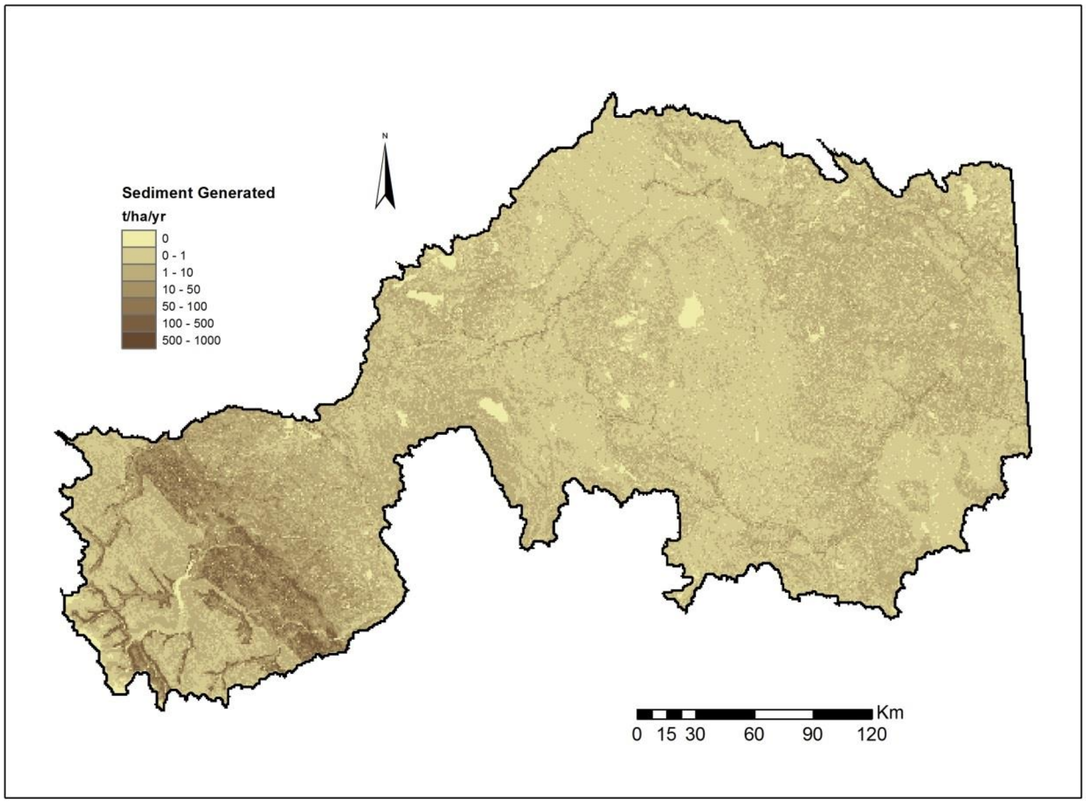

Figure G. Sediment generated per 64 ha in the North Saskatchewan and Battle River Watersheds in Alberta, Canada. Sediment generation is based on the Revised Universal Soil Loss Equation.

4. Stream Flow & Tracking Loads

Once raindrop agents have reached a river cell, all of the water flow and quality information they contain (flow, nutrient load, and sediment load) are transferred to the river network. Because each node in the river network is connected to its upstream and downstream neighbours, water flow and loading information is transferred downstream, such that each cell in the river network is able to track the total, cumulative amount of flow and loading that passes through that point (Figure H). Monitoring stations that have been set up along the river network track the same metrics; this allows for easy export of data from these points of interest.

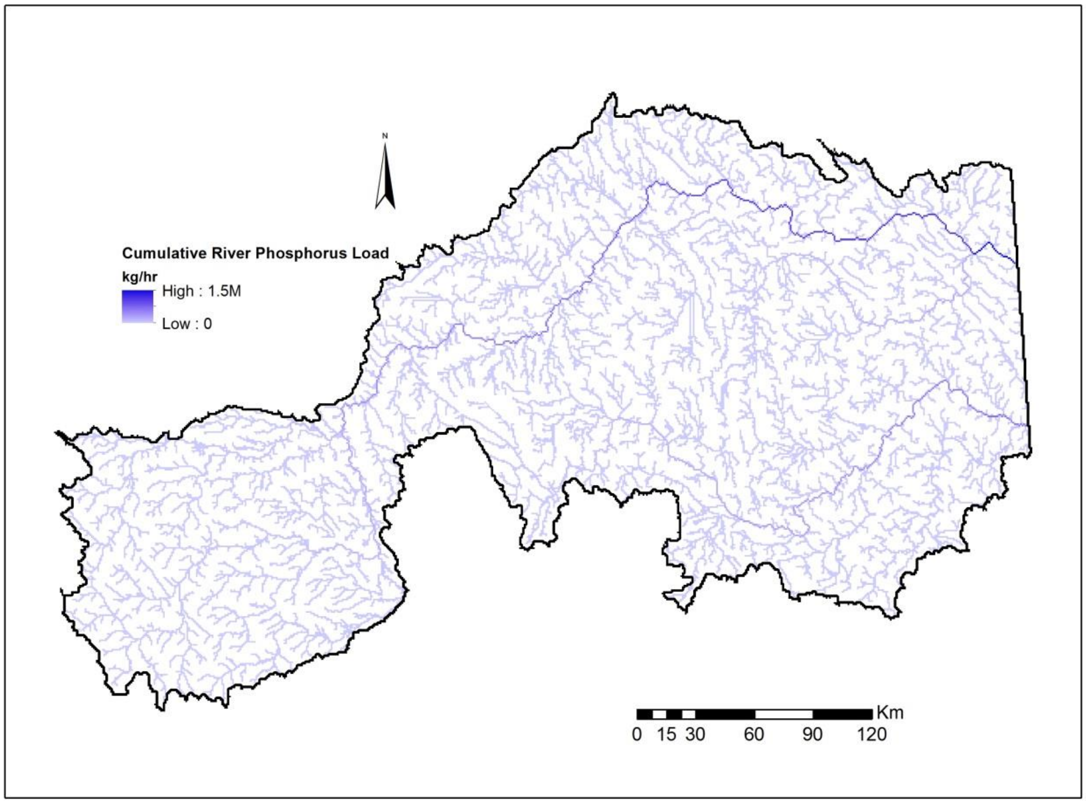

Figure H. Cumulative phosphorus load throughout the river network in the North Saskatchewan and Battle River Watersheds in Alberta, Canada.

5. Source and Sink Areas

Once surface and stream flow are complete, several metrics are calculated for each cell in the study region. Each raindrop agent keeps track of its origin cell and its route through other downslope patches until it reaches the river network. Therefore, the final amount of pollutant load in each raindrop, after it has completed its flow to the river, is mapped back to its origin cell, providing a map of actual pollutant supply from each cell (Figure I). Combining the phosphorus supply and retention maps provides an estimate of the net contribution of a given cell to the phosphorus reaching the river network. Combining the supply map with the retention map (Figure F), each cell's net contribution can be calculated by subtracting the amount of each pollutant retained from each's cells pollutant supply (Figure J).

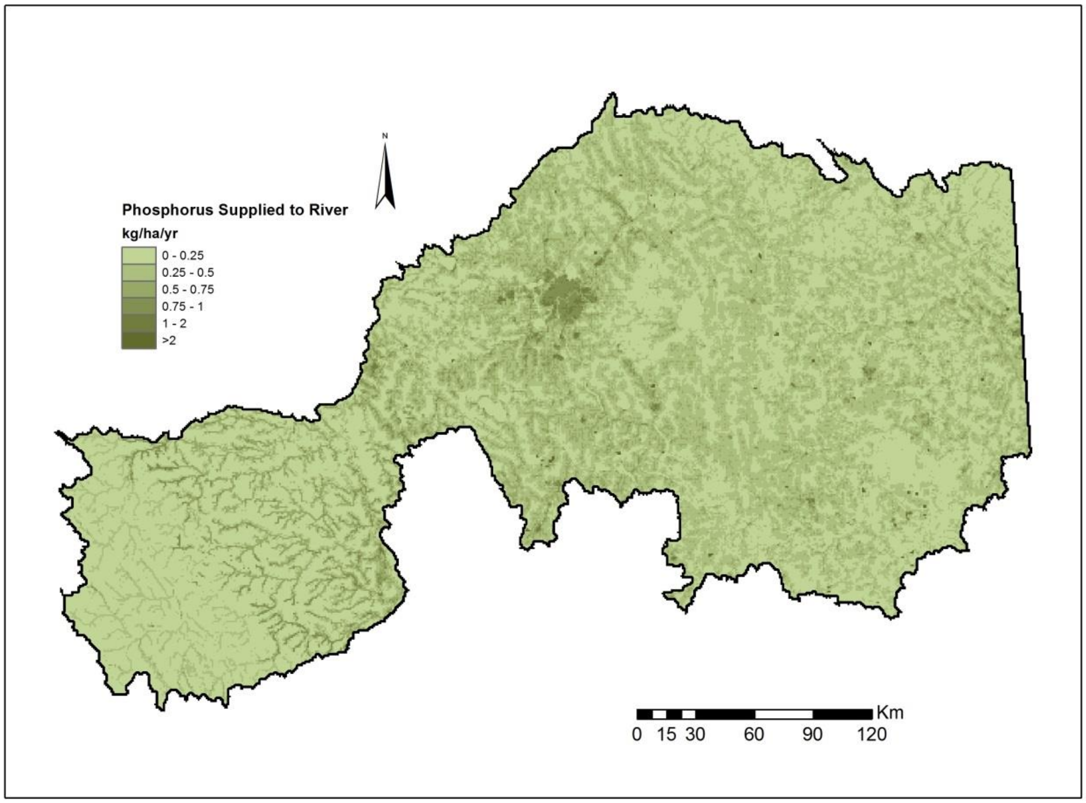

Figure I. Phosphorus supplied to the river network, after accounting for phosphorus retained during overland flow.

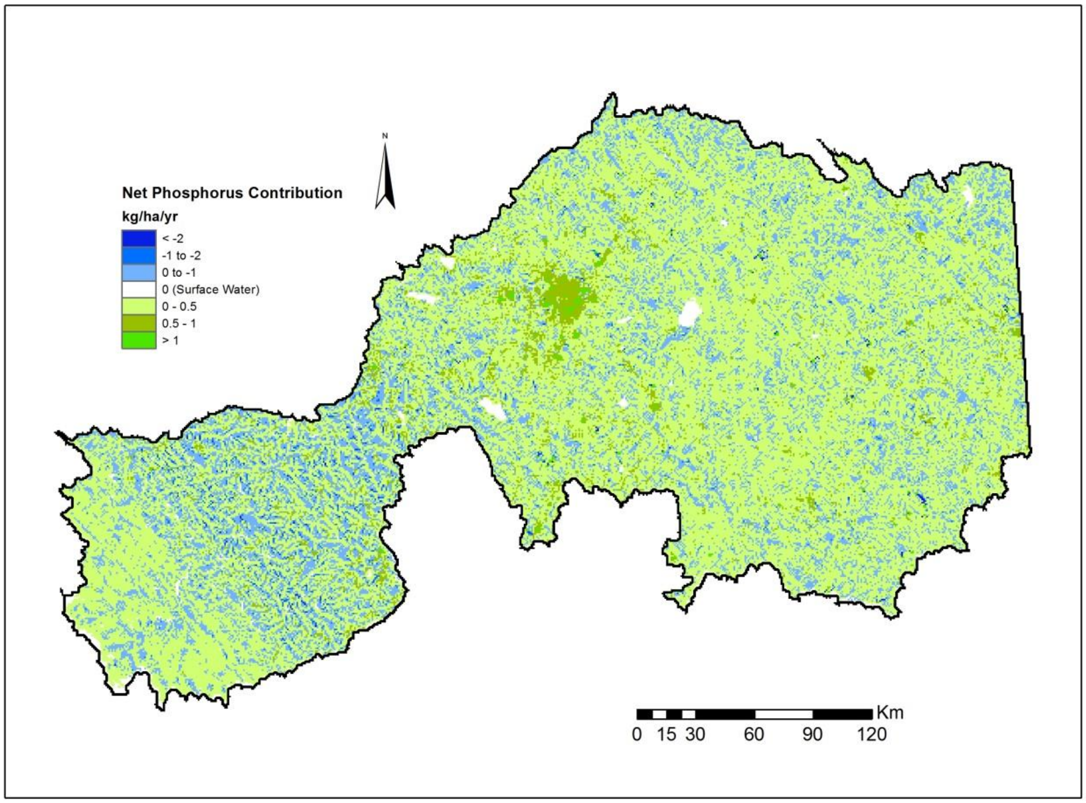

Figure J. Net phosphorus per 64 ha in the North Saskatchewan and Battle River Watersheds in Alberta, Canada.

Water Model Calibration

We calibrated the water model based on cumulative annual phosphorus loading data obtained for 8 points in the headwaters of the North Saskatchewan watershed. We conducted a global calibration by varying the export coefficients; we obtained 10 parameter sets using Latin hypercube sampling, and the parameter set that performed best for each natural region. After this procedure, the Spearman's Rank correlation between the calibrated model results and observed phosphorus loads at the 8 monitoring points was 0.33.

Table I. Variables used in the Water Purification Model.

Variable Name	Description	Units
Water Purification Model		
Global variables and model control		
**headwaters**	Set of river agents with no children (i.e. most upstream river agents)	Set of river agents
**tributaries**	Temporary agentset containing river agents iteratively downstream of the previous "tributary-parents", used to transport information down the river network	Set of river agents
**tributary-parents**	Temporary agentset of river agents immediately downstream of "tributaries"	Set of river agents
Water loading categories		
**general-ag**	Annual crops (includes ag-qs and lc_120)	hectares
**barren**	Unvegetated, non-human footprint (lc_31-33)	hectares
**cutblock**	Forestry cutblocks (equal to ag-for)	hectares
**feedlots**	confined livestock feeding operations	hectares
**hard-roads**	All paved and gravel roads (hard-qs)	hectares
**industrial**	Oil/gas plants, windmills, forestry mills (subset of urbind-qs)	hectares
**mines-disturbed**	Disturbed open-pit mines, peat mines (subset of urbind-qs)	hectares
**mines-undisturbed**	Undisturbed areas within mine sites (subset of urbind-qs)	hectares
**pasture**	Hay and tame pasture (equivalent to pasture10)	hectares
**pipelines**	Oil/gas pipelines (subset of soft-qs)	hectares
**recreation**	Golf courses, ski resorts, etc. (subset of urbind-qs)	hectares
**seismic**	Seismic lines (subset of soft-qs)	hectares
**trails-motorized**	ATV & truck trails, winter roads (subset of soft-qs)	hectares
**trails-nonmotorized**	Vegetated road and railway verges (subset of soft-qs)	hectares
**transmission-lines**	Electrical transmission lines/right-of-ways (subset of soft-qs)	hectares
**urban**	Urban areas (subset of urbind-qs)	hectares
**wellpads**	Oil/gas wellpads (subset of urbind-qs)	hectares
**XX-load-n**	Nitrogen entering a cell's runoff originating from water loading category XX (from above list)	kg
**XX-load-p**	Phosphorus entering a cell's runoff originating from water loading category XX (from above list)	kg
**XX-load-tss**	TSS entering a cell's runoff originating from water loading category XX (from above list)	kg
Water landscape characteristics		

**basin**	Integer ID for watershed a cell is within	Categorical
**is-station**	True/false indicating if a water monitoring stating is located within this cell (Water Model)	True/false
**elevation**	Elevation based on a DEM	m
**infiltration**	Amount of preciptiation that infiltrates into soil	mm
**channel-elevation**	Channel elevation data from channel-dataset applied to patches. A more refined elevation that better captures channel features	m
**rainfall**	Annual precipitation	mm
**k-factor**	soil erodibility used in RUSLE	n/a
**ls-factor**	slope length factor used in RUSLE	n/a
**c-factor**	cover factor used in RUSLE	n/a
**hydro-elevation**	Modified elevation data derived by averaging elevation and channel-elevation (to represent elevation of water column)	m
**slope**	slope calculated from elevation data	%
**runoff-coefficient**	percent of rainfall that leaves a cell via surface runoff	%
**hillslope-sediment-generated**	eroded sediment generated at each cell calculated using RUSLE	tonnes
**is-river**	True/false indicating if a river crosses a cell	true/false
**outlet-id**	Code indicating if a river outlet is located in a cell (o if no outlet)	categorical
**removal-rate**	Percent of nutrients removed on each cell during overland water flow	%
**runoff**	surface runoff from a cell	mm
**flow**	Volume of water currently on a cell	m^3
**load-n**	Total nitrogen released in surface runoff on a cell	kg
**load-p**	Total phosphorus released in surface runoff on a cell	kg
**load-tss**	Total tss released in surface runoff on a cell	kg
**load-n-perha**	Nitrogen released as runoff per hectare	kg/ha
**load-p-perha**	Phosphorus released as runoff per hectare	kg/ha
**load-tss-perha**	TSS released as runoff per hectare	kg/ha
**n-deposited**	Nitrogen originating from upslope that is retained by a cell during overland flow	kg
**p-deposited**	Phosphorus originating from upslope that is retained by a cell during overland flow	kg
**tss-deposited**	TSS originating from upslope that is retained by a cell during overland flow	kg
**sediments-deposited**	Sediments originating from upslope that is retained by a cell during overland flow	tonnes
**n-supply**	Nitrogen originating from a cell that eventually reaches the	kg

	river network (i.e. is not retained downslope). Subset of n-load.	
**p-supply**	Phosphorus originating from a cell that eventually reaches the river network (i.e. is not retained downslope). Subset of p-load.	kg
**tss-supply**	TSS originating from a cell that eventually reaches the river network (i.e. is not retained downslope). Subset of tss-load.	kg
**sediment-supply**	Sediment originating from a cell that eventually reaches the river network (i.e. is not retained downslope). Subset of hillslope-sediment-generated.	tonnes
**net-n**	Net contribution of a cell to nitrogen reaching the river network. Calculated as n-supply minus n-deposited	kg
**net-p**	Net contribution of a cell to phosphorus reaching the river network. Calculated as p-supply minus p-deposited	kg
**net-tss**	Net contribution of a cell to TSS reaching the river network. Calculated as tss-supply minus tss-deposited	kg
**net-sediment**	Net contribution of a cell to sediment reaching the river network. Calculated as sediment-supply minus sediments-deposited	tonnes
**cum-river-n**	Cumulative annual nitrogen within the river on a given patch (0 if no river present)	kg
**cum-river-p**	Cumulative annual phosphorus within the river on a given patch (0 if no river present)	kg
**cum-river-tss**	Cumulative annual TSS within the river on a given patch (0 if no river present)	kg
**cum-river-sediment**	Cumulative annual sediment within the river on a given patch (0 if no river present)	tonnes
**cum-river-flow**	Cumulative annual water flow within the river on a given patch (0 if no river present)	m^3
**Water Model Agent Variables**		
**Raindrop agents**		
**flow-volume**	Volume of water contained in a raindrop agent	m^3
**sediment-mass**	Mass of sediment contained in a raindrop agent	tonnes
**p-mass**	Mass of phosphorus contained in a raindrop agent	kg
**n-mass**	Mass of nitrogen contained in a raindrop agent	kg
**tss-mass**	Mass of TSS contained in a raindrop agent	kg
**my-home**	Cell where each raindrop originated (i.e. fell as precipitation)	single cell
**my-route**	Collection of cells traversed by a raindrop during overland flow	set of cells

**River Agents**		
**parent**	Adjacent linked river agents immediately upstream of a river	set of river agents
**children**	All adjacent linked river agents immediately downstream of a river	set of river agents
**is-outlet**	True/false indicating if a river agent is at the outlet of the river	True/false
**my-flow**	Total flow passing through a river agent	m^3
**my-N**	Cumulative mass of nitrogen in a river	kg
**my-P**	Cumulative mass of phosphorus in a river	kg
**my-tss**	Cumulative mass of TSS in a river	kg
**my-sediment**	Cumulative mass of sediment in a river	tonnes
**N-to-contribute**	Mass of N that will flow downstream	kg
**P-to-contribute**	Mass of P that will flow downstream	kg
**tss-to-contribute**	Mass of TSS that will flow downstream	kg
**sediment-to-contribute**	Mass of sediment that will flow downstream	tonnes
**flow-to-contribute**	Water that will flow downstream	m^3
**Station agents**		
**station-name**	Descriptive name/location of water monitoring point	Text
**my-monitoring-point**	Cell containing a river where a monitoring station is located	single cell
**station-flow**	Cumulative annual water flowing past a station	m^3
**station-N**	Cumulative mass of N flowing past a station	kg
**station-P**	Cumulative mass of P flowing past a station	kg
**station-TSS**	Cumulative mass of TSS flowing past a station	kg
**station-sediment**	Cumulative mass of sediment flowing past a station	tonnes
